# Accelerated T2-Weighted TSE Imaging of the Prostate Using Deep Learning Image Reconstruction: A Prospective Comparison with Standard T2-Weighted TSE Imaging

**DOI:** 10.3390/cancers13143593

**Published:** 2021-07-17

**Authors:** Sebastian Gassenmaier, Saif Afat, Marcel Dominik Nickel, Mahmoud Mostapha, Judith Herrmann, Haidara Almansour, Konstantin Nikolaou, Ahmed E. Othman

**Affiliations:** 1 Department of Diagnostic and Interventional Radiology, Eberhard-Karls-University Tuebingen, Hoppe-Seyler-Straße 3, 72076 Tuebingen, Germany; sebastian.gassenmaier@med.uni-tuebingen.de (S.G.); saif.afat@med.uni-tuebingen.de (S.A.); judith.herrmann@med.uni-tuebingen.de (J.H.); haidara.al-mansour@med.uni-tuebingen.de (H.A.); konstantin.nikolaou@med.uni-tuebingen.de (K.N.); 2 MR Applications Predevelopment, Siemens Healthcare GmbH, 91052 Erlangen, Germany; marcel.nickel@siemens-healthineers.com; 3 Digital Technology & Innovation, Siemens Medical Solutions USA, Inc., Princeton, NJ 08540, USA; mahmoud.mostapha@siemens-healthineers.com; 4 Cluster of Excellence iFIT (EXC 2180) “Image Guided and Functionally Instructed Tumor Therapies”, University of Tuebingen, 72076 Tuebingen, Germany; 5 Department of Neuroradiology, University Medical Centre, Johannes Gutenberg University Mainz, 55131 Mainz, Germany

**Keywords:** deep learning, multiparametric magnetic resonance imaging, prostatic neoplasms, diagnostic imaging

## Abstract

**Simple Summary:**

Multiparametric magnetic resonance imaging (mpMRI) has become an important diagnostic tool in the assessment of clinically significant prostate cancer. The disadvantages of this technique are primarily related to its long examination time and limited availability. This prospective study presents a novel accelerated deep learning image reconstructed T2-weighted turbo spin echo (TSE) sequence providing an acquisition time reduction of more than 60%. The study results show that the acquisition of three imaging planes of the prostate is feasible within 3:50 min, using deep learning image reconstruction, compared to 10:21 min in standard imaging. Additionally, image quality parameters were evaluated to be superior in deep learning imaging.

**Abstract:**

Multiparametric MRI (mpMRI) of the prostate has become the standard of care in prostate cancer evaluation. Recently, deep learning image reconstruction (DLR) methods have been introduced with promising results regarding scan acceleration. Therefore, the aim of this study was to investigate the impact of deep learning image reconstruction (DLR) in a shortened acquisition process of T2-weighted TSE imaging, regarding the image quality and diagnostic confidence, as well as PI-RADS and T2 scoring, as compared to standard T2 TSE imaging. Sixty patients undergoing 3T mpMRI for the evaluation of prostate cancer were prospectively enrolled in this institutional review board-approved study between October 2020 and March 2021. After the acquisition of standard T2 TSE imaging (T2_S_), the novel T2 TSE sequence with DLR (T2_DLR_) was applied in three planes. Overall, the acquisition time for T2_S_ resulted in 10:21 min versus 3:50 min for T2_DLR_. The image evaluation was performed by two radiologists independently using a Likert scale ranging from 1–4 (4 best) applying the following criteria: noise levels, artifacts, overall image quality, diagnostic confidence, and lesion conspicuity. Additionally, T2 and PI-RADS scoring were performed. The mean patient age was 69 ± 9 years (range, 49–85 years). The noise levels and the extent of the artifacts were evaluated to be significantly improved in T2_DLR_ versus T2_S_ by both readers (*p* < 0.05). Overall image quality was also evaluated to be superior in T2_DLR_ versus T2_S_ in all three acquisition planes (*p* = 0.005–<0.001). Both readers evaluated the item lesion conspicuity to be superior in T2_DLR_ with a median of 4 versus a median of 3 in T2_S_ (*p* = 0.001 and <0.001, respectively). T2-weighted TSE imaging of the prostate in three planes with an acquisition time reduction of more than 60% including DLR is feasible with a significant improvement of image quality.

## 1. Introduction

Multiparametric magnetic resonance imaging (mpMRI) of the prostate has become the standard of care in prostate cancer imaging during the last decade [1,2]. mpMRI plays an important role in biopsy planning, local staging, as well as active surveillance [2,3,4,5,6]. The technical development and increasing experience with mpMRI reporting has finally led to the establishment of the prostate imaging reporting and data system (PI-RADS). The currently recommended protocol of the European Society of Urology consists of the following sequences: T2-weighted turbo spin echo (TSE) imaging in three planes with a slice thickness of 3 mm without gaps and with high morphological resolution. Furthermore, diffusion-weighted imaging (DWI) including apparent diffusion coefficient maps (ADC) as well as dynamic contrast-enhanced imaging should be acquired, to provide further parameters for analysis. Additionally, a precontrast T1-weighted sequence of the pelvis with a larger field of view should be acquired, for lymph node staging as well as for assessment of pelvic bones. A disadvantage of this comprehensive protocol consists of rather long acquisition times, ranging approximately from 30 to 45 min. This is especially problematic as prostate cancer commonly affects elderly men who may have difficulties remaining motionless during long MRI examinations. Furthermore, long examination times are problematic due to the growing demand for mpMRI of the prostate, resulting from extended life expectancy on the one hand and increasing importance of the examination modality to prevent significant prostatic cancers on the other hand. Especially the role in active surveillance might determine a significant contribution for increasing demands regarding mpMRI in future.

Established methods to shorten the acquisition time include parallel imaging techniques in TSE imaging or compressed sensing (CS) in gradient echo imaging. However, a significant disadvantage of parallel imaging is the loss of signal-to-noise ratio (SNR) proportional to the square root of the scan acceleration. Therefore, in clinical protocols, parallel imaging acceleration does usually not exceed a factor of two for two-dimensional acquisitions and a factor of four for three-dimensional acquisitions.

One of the newest techniques applied for MRI acquisition acceleration is based on deep learning (DL). While DL techniques have been primarily applied to facilitate and support diagnosis so far, recent applications concentrate on the acceleration of MRI protocols [7,8,9,10,11]. In previous studies, it could be shown that DL, e.g., via variational networks, is able to significantly accelerate MRI protocols of the abdomen, knee or of the pituitary [12,13,14,15]. However, literature regarding the clinical impact of DL strategies in accelerated MRI protocols is still sparse.

Due to the increasing importance of prostate MRI that is hampered by the long examination times, a thorough, systematic investigation of the potential benefits of DL reconstruction techniques in mpMRI seems worthwhile. Therefore, the aim of this study was to investigate the impact of DL reconstruction in accelerated T2 TSE imaging of the prostate in three orthogonal planes on image quality, lesion conspicuity, and diagnostic confidence, compared to standard T2 TSE imaging.

## 2. Materials and Methods

### 2.1. Study Design

**Figure 1:**
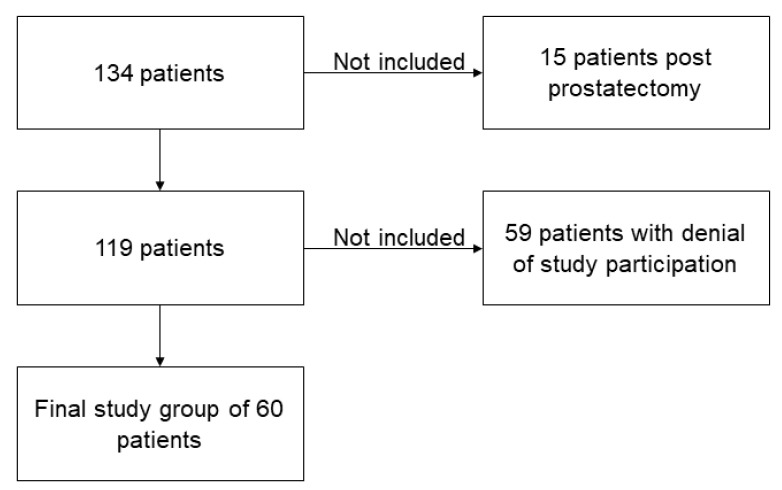
Flowchart of study participants.

This prospective study was approved by the institutional review board. Inclusion criteria were: examination for evaluation of prostate cancer on appropriate 3T MRI scanners with installed DL reconstruction algorithm. Exclusion criteria were: non-conditional implants, severe claustrophobia, and status post prostatectomy. Figure 1 shows the flowchart of this study (Figure 1). Sixty patients who underwent 3T MRI of the prostate for evaluation of carcinoma between October 2020 and March 2021 were finally included (Table 1). Study participation was voluntary, and all patients gave informed consent to participate in this study. All study procedures were in line with the declaration of Helsinki and its later amendments.

### 2.2. Deep Learning Image Reconstruction Technique

An unrolled variational network was employed for reconstruction [16,17]. Inspired by the iterative optimization used in CS, the trainable network alternated between data consistency steps and image regularization steps using a convolutional network. From this viewpoint, the approach can be considered a natural development from CS in that the regularization is not postulated but instead determined on representative data. Similar to CS, the input to the network consists of undersampled k-space data, coil sensitivity maps estimated from reference lines, and for the network used in the present study also a normalization field for image homogenization. Conventional sampling patterns also used in parallel imaging were used for acceleration. As the reconstruction was not designed to modify the image contrast, but instead mainly focused on signal-to-noise enhancement, this procedure had the additional advantage that the effect of acquisition parameters, such as echo time, repetition time, and echo train length was identical to conventional reconstructions.

The parameters of the model were determined through supervised training using about 10,000 slices from volunteer TSE acquisitions on various clinical 1.5T and 3T scanners (MAGNETOM scanners, Siemens Healthcare). The fully sampled training data was acquired in different body regions (head, pelvis, and knee) and with different image contrasts. The input data was gained via retrospective downsampling by a factor of 4 and usage of a phase resolution of 75%. An L1-norm and multiscale version of the structural similarity (SSIM) content between network prediction and ground truth images encompassed the loss function. The training was integrated in PyTorch and performed on a commercially available GPU cluster with 32 GB of memory. Finally, the trained network was converted for use in a scanner-integrated inference framework. Inference time for a single slice in the actual deployment was about 3 s for CPU on average and 0.5 s for GPU.

### 2.3. Imaging Protocol

All MRI studies were performed in clinical routine using three different scanners (MAGNETOM Skyra, MAGNETOM Prisma^fit^, and MAGNETOM Vida, Siemens Healthcare). Butylscopolamine bromide was applied prior to the examination, given no present contraindications (e.g., cardiac arrhythmia or glaucoma). Patients were scanned in dorsal position using a setup of 12 elements of a 32-channel spine coil as well as an 18-channel body coil. No endorectal coil was applied in our imaging center. The institution’s standard mpMRI protocol consisted of the following sequences: T2w TSE imaging in three planes; DWI with three different acquired b-values in axial plane (50 s/mm², 500 s/mm², and 1000 s/mm²) as well as one calculated b-value of 2000 s/mm² and ADC mapping; for evaluation of possible bone lesions and lymph node status, a T1w precontrast TSE imaging with a larger field of view was obtained. Furthermore, after application of contrast media (0.1 mmol/kg body weight gadobutrol; Gadovist, Bayer Healthcare) using a flow rate of 1.5 mL/s followed by a saline flush of 20 mL, dynamic contrast-enhanced gradient echo imaging was acquired in axial direction followed by an additional post-contrast gradient-echo axial sequence.

After completion of standard T2w TSE imaging (T2_S_), the novel T2w TSE imaging with deep learning image reconstruction (T2_DLR_) was acquired using a prototype sequence.

Imaging parameters are displayed in Table 2. Acquisition time of axial T2_S_ was 4:37 min as compared to 1:38 min of T2_DLR_ (65% reduction of acquisition time), of coronal T2_S_ was 3:07 min versus 1:10 min of T2_DLR_ (63% reduction of acquisition time), and of sagittal T2_S_ was 2:37 min versus 1:02 min of T2_DLR_ (61% reduction of acquisition time). Overall, acquisition time for T2_S_ resulted in 10:21 min versus 3:50 min for T2_DLR_.

### 2.4. Image Analysis

Image analysis was performed independently by two radiologists with five and eight years of experience. Reading sessions were carried out in a blinded random order consisting of datasets of T2 imaging (DLR or standard), dynamic contrast-enhanced imaging, as well as DWI, including ADC maps. One hundred and twenty imaging sets were created (60 datasets with T2_S_ and 60 datasets with T2_DRL_). Both readers were blinded to the description of sequence names, to patient data as well as to the official radiological report.

T2 score and PI-RADS score according to PI-RADS v.2.1 were assessed for all datasets. The exact location and lesion size were reported for the most suspicious lesions given a PI-RADS score ≥ 3.

Additionally, image quality parameters were assessed for the following items using an ordinal Likert scale ranging from 1 to 4 (1 = non-diagnostic; 4 = excellent): noise levels, lesion conspicuity, magnitude of artifacts, diagnostic confidence of the readers and image quality overall.

Furthermore, T2_S_ and T2_DLR_ sequences of each patient were presented to both radiologists mentioned above and were evaluated regarding the naturality of image impression using a Likert scale ranging from 1 to 4 (1 = unnatural; 4 = very natural).

### 2.5. Statistical Evaluation

Statistical evaluation was performed using commercially available statistical software (SPSS Statistics Version 26; IBM). Continuous variables are shown using mean ± standard deviation (std), ordinal scaled variables are shown using median and interquartile range (IQR). The Wilcoxon signed-rank test was used for paired data of ordinal structure and non-normally-distributed parametric variables. *p*-values were adjusted using Bonferroni procedure. Intra- and inter-reader variability were assessed using Cohen’s kappa. The significance level alpha was set at 0.05.

## 3. Results

### 3.1. Patients’ Characteristics

The mean patient age was 69 ± 9 years (range, 49–85 years). The median prostate specific antigen (PSA) level was 7.2 ng/mL (IQR 5.9–9.7) (Table 1).

The results of the more experienced reader 1 are described in the following. All results are available within the tables of this manuscript.

### 3.2. Evaluation of Qualitative Imaging Parameters

Analysis of the inter-reader agreement between both readers regarding image quality parameters using Cohen’s kappa resulted in 0.76 for T2_S_ and 0.79 for T2_DLR_. The impact and extent of image noise was rated to be significantly less in T2_DLR_ compared to T2_S_ with the following ratings: median of 4 (IQR 4–4) for T2_DLR_ versus a median of 3 (IQR 3–3) for T2_S_ in axial imaging, a median of 4 (IQR 4–4) for T2_DLR_ versus a median of 3 (IQR 3–3) for T2_S_ in coronal imaging, and a median of 4 (IQR 3–4) for T2_DLR_ versus a median of 3 (IQR 3–3) for T2_S_ in sagittal imaging (all *p* < 0.001). The extent of artifacts was also rated to be less in T2_DLR_ versus T2_S_ in axial imaging with a median of 4 (IQR 4–4) compared to 3 (3–4) (*p* = 0.003). The extent of artifacts in T2_DLR_ was also significantly less in coronal (*p* = 0.002) and sagittal imaging (*p* = 0.002). There was no significant difference regarding the natural appearance of both sequences in all planes with a median of 4 for T2_S_ and T2_DLR_ (*p* > 0.05). Overall image quality was rated higher in axial T2_DLR_ (median of 4 (IQR 4–4)) as compared to T2_S_ (median of 3 (IQR 3–4)) (*p* < 0.001). Similar results regarding image quality were obtained for T2_DLR_ versus T2_S_ in coronal (*p* = 0.002) and sagittal imaging (*p* = 0.002). Diagnostic confidence was evaluated to be higher in T2_DLR_ with a median of 4 (4–4) as compared to T2_S_ with a median of 4 (3–4) (*p* = 0.03). Table 3 shows the image quality results of both readers.

### 3.3. PI-RADS Scoring and Lesion Conspicuity

**Figure 2:**
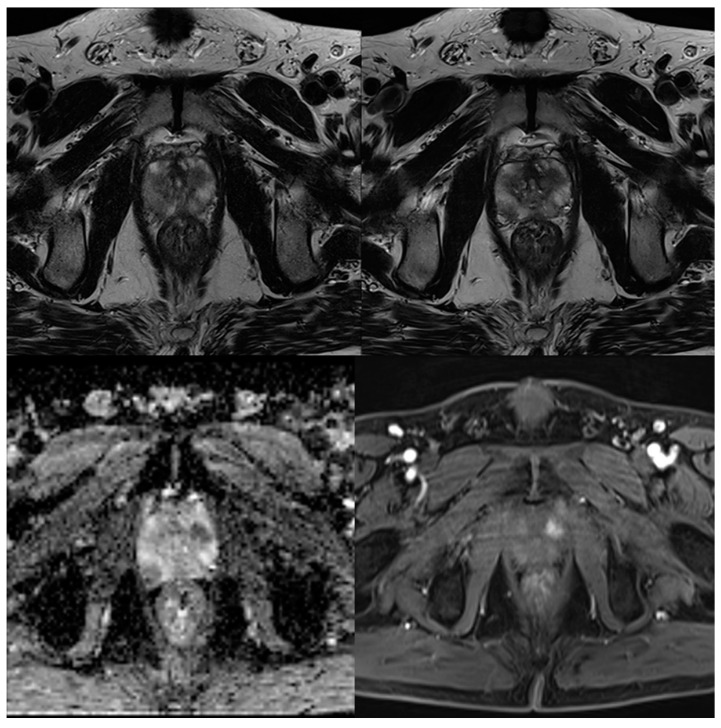
A 59-year-old male patient with suspicion of prostate cancer. Example of axial standard T2-weighted TSE imaging (T2_S_) on the top left-hand side and deep learning–reconstructed (T2_DLR_) imaging on the top right-hand side. The bottom row shows apparent diffusion coefficient (ADC) map on the left-hand side and dynamic contrast-enhanced (DCE) imaging on the right-hand side. PI-RADS 5 lesion in the transition zone was found by both readers in both sequences. Motion artifacts are especially reduced in T2_DLR_ demonstrating the advantages of reduced acquisition time.

**Figure 3:**
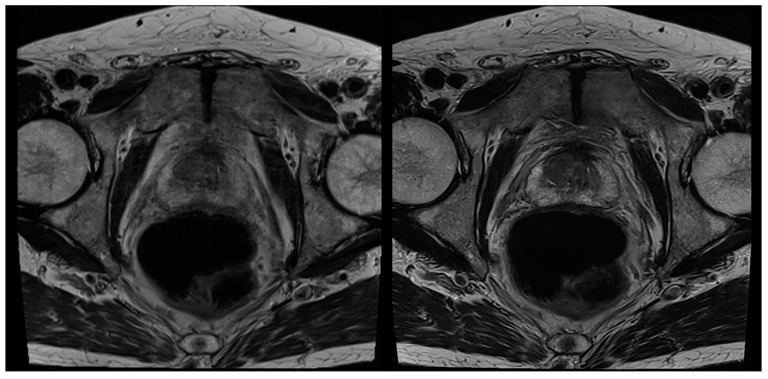
A 72-year-old male patient with suspicion of prostate cancer. Example of axial standard T2-weighted TSE imaging (T2_S_) on the left-hand side and deep learning–reconstructed (T2_DLR_) imaging on the right-hand side. Similar to Figure 2, less motion artifacts occurred in T2_DLR_ with sharper depiction of the prostate.

**Figure 4:**
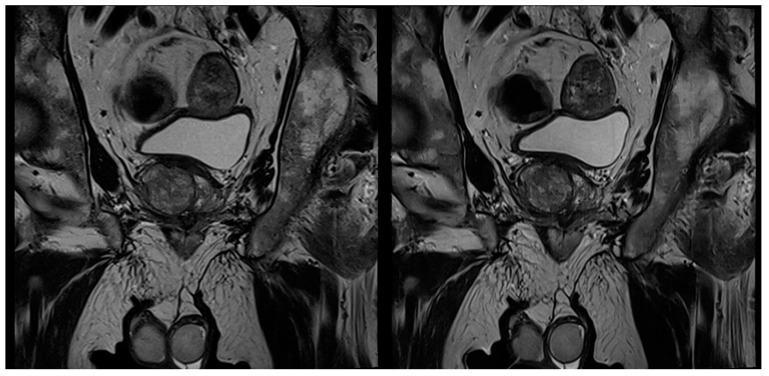
A 67-year-old male patient with suspicion of prostate cancer. Example of coronal standard T2-weighted TSE imaging (T2_S_) on the left-hand side and deep learning–reconstructed (T2_DLR_) imaging on the right-hand side. Advantages of T2_DLR_ are demonstrated, regarding less image noise and improved delineation of anatomic structures.

**Figure 5:**
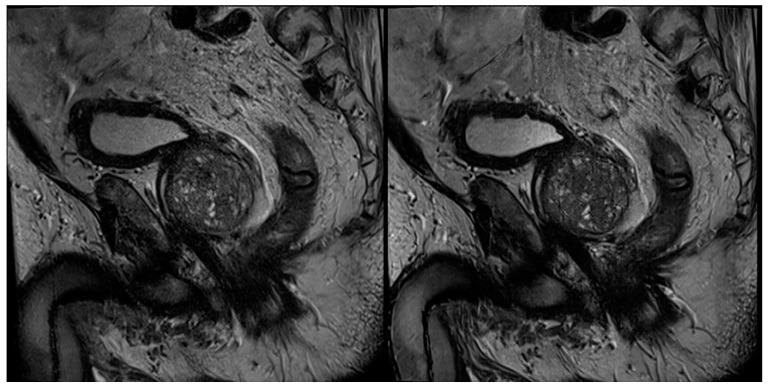
A 76-year-old male patient with suspicion of prostate cancer. Example of sagittal standard T2-weighted TSE imaging (T2_S_) on the left-hand side and deep learning–reconstructed (T2_DLR_) imaging on the right-hand side. This figure demonstrates the advantages of deep learning image reconstruction, regarding image noise and sharpness of organ structures.

In no case was a different scoring affecting patient management (<3 versus ≥3) found between the readers and between the sequences. Table 4 shows the detailed results of the T2 and PI-RADS scoring of both readers. In 39 cases, a PI-RADS score ≥3 was found as the most suspicious lesion. In eight of these 39 cases, the most suspicious lesions were found in the transition zone, and in 31 cases in the peripheral zone. Inter-reader agreement regarding T2-scoring was 0.823 for T2_S_ and 0.932 for T2_DLR_ (Table 5). Cohen’s kappa for inter-reader agreement of PI-RADS scoring was 0.905 for T2_S_ and 0.946 for T2_DLR_ (Table 5). Lesion conspicuity was rated superior in T2_DLR_ by both readers compared to T2_S_ with a median of 4 (4–4) versus a median of 3 (3–4) for both readers (*p* = 0.001 and *p* < 0.001, respectively). There was no significant difference between lesion size measurements between T2_S_ and T2_DLR_ (Table 6). Figure 2, Figure 3, Figure 4 and Figure 5 show examples of T2_S_ and T2_DLR_.

## 4. Discussion

This prospective study investigated the feasibility and impact of DL image reconstruction in accelerated T2w TSE imaging of the prostate in three planes. The results demonstrate that the application of DL image reconstruction in T2-weighted TSE imaging of the prostate is robust and ready to be clinically implemented, as well as being able to achieve a reduction of acquisition time above 60% and superior image quality, compared to a standard T2 acquisition. No significant difference between PI-RADS scoring and T2 scoring could be observed between the sequences.

The potential of DL techniques has been previously demonstrated in several studies [18,19,20,21]. However, most studies have primarily investigated DL methods regarding diagnosis support, e.g., regarding the detection of cancer or analysis of lung tissue [8,18,22]. It was shown in recent studies on prostate MRI that DL can be applied for cancer detection and classification and also for registration with histopathological images [10,11,23,24]. Wang et al. demonstrated that DL could also be applied for the omission of endorectal coils in mpMRI without compromising the image quality regarding noise [25]. However, one of the most important recent developments regarding DL is related to MRI acquisition times. DL-based reconstructions can be used for the acceleration of MRI sequences via the increased undersampling of the acquired data, e.g., via a variational neural network [16]. The clinical applicability of this technique was already demonstrated in knee MRI examinations by Recht et al. [13]. However, in this study, the authors used retrospectively undersampled data. Meanwhile, the potential of DL-based reconstructions for the acceleration and/or improvement of image quality has also been demonstrated in studies with “real” undersampled data, e.g., in prostate MRI and MRI of the pituitary gland [12,17]. However, in a previous prostate DL MRI investigation only a small number of patients were examined in only one imaging plane, limiting the generalizability of the results [17]. The present study demonstrates the first systematic prospective approach with the analysis of three imaging planes in a much larger patient cohort.

Conventional methods for shortening acquisition times involve parallel imaging or CS [26,27,28,29]. Although by using parallel imaging, a drastic reduction of acquisition time is theoretically possible, in clinical routine only factors of 2–4 are routinely applied. The reasons for this are due to SNR loss proportional to the square root of the scan acceleration and typical artifacts as noise bands [30]. CS is an established method for MRI acquisition acceleration that is based on redundancy of imaging data; however, a common disadvantage of this technique is the unnatural appearance of these images, as well as the long processing time [26]. In the present study, the DL-reconstructed images were evaluated as superior, regarding both image quality and noise. The very low degree of noise in some T2_DRL_ images may cause an unrealistic image perception. Despite this issue, both readers evaluated the appearance of T2_DLR_ images to be very natural, without significant difference to the T2_S_.

Despite the drastic shortening of acquisition time and improved image quality, the question arises as to which clinical benefits might be drawn from these results. MRI of the prostate has undergone an extraordinary evolution during the last decade, nowadays leading to a structured and standardized analysis via the PI-RADS classification system [2]. mpMRI is an important tool for biopsy planning and also for prostate cancer detection [4,31]. Furthermore, in the future, mpMRI might play an important role for patients under active surveillance [32]. However, an increase in demand for an examination does not automatically lead to an increase in supply, due to the restricted availability of MRI scanners and comparably long examination times using standard techniques. This is especially problematic due to the long examination times necessary for mpMRI of the prostate, to align with current guidelines and to achieve sufficient image and report quality. Therefore, the demonstrated approach of a drastic reduction of T2 TSE acquisition time above 60% provides enormous potential for the increase of scanner availability. T2 TSE imaging in three planes in less than 4 min might even allow ultra-short screening protocols for prostate cancer to be created. This is even more important, due to the updated PI-RADS version 2.1 with the possibility of acquiring only two planes of T2 TSE imaging and further research regarding biparametric MRI with omission of dynamic contrast-enhanced imaging [33].

### Limitations

This study investigated only DL reconstructions of T2w TSE imaging. No other sequences were reconstructed using DL technology. However, due to the time-consuming acquisition process of high-resolution morphological T2 images, this sequence was preferred for initial DL implementation. Further studies will be necessary to investigate the impact on other sequences. Furthermore, it is possible to change the image perception via a change of DL networks or artificial increase of noise (dithering). However, these approaches were beyond the scope of our study and, therefore, not further analyzed. Additionally, as many patients were examined in an outpatient setting, possible biopsy results were not available and therefore diagnostic accuracy regarding histopathologic correlation was not part of this study.

## 5. Conclusions

This prospective study demonstrates the potential of a new DL reconstruction algorithm in accelerated T2w TSE imaging of the prostate in three planes with a reduction of acquisition times above 60% combined with an improvement of image quality and a reduction of artifacts, compared to standard T2w imaging. Concluding, DL reconstructions may be of great importance for establishing ultrafast screening protocols in prostate MRI.

## Figures and Tables

**Table 1:** Patients’ characteristics.

Characteristics	Values
Number of patients	*n* = 60
Age, mean ± standard deviation	69 ± 9 years
Sex	100% male
PSA, median (interquartile range)	7.2 ng/mL (5.9–9.7 ng/mL)

**Table 2:** MRI acquisition parameters.

	Axial		Coronal		Sagittal	
	T2_S_	T2_DLR_	T2_S_	T2_DLR_	T2_S_	T2_DLR_
TR (ms)	4470	4470	7480	7760	7480	6900
TE (ms)	104	104	101	101	101	101
Averages	3	1	3	1	2	1
Voxel size (mm)	0.3 × 0.3 × 3.0	0.3 × 0.3 × 3.0	0.3 × 0.3 × 3.0	0.3 × 0.3 × 3.0	0.3 × 0.3 × 3.0	0.3 × 0.3 × 3.0
Field of view (mm)	200	200	200	200	200	200
Slice thickness (mm)	3	3	3	3	3	3
Parallel imaging factor	3	3	3	3	2	3
Acquisition time (min:sec)	4:37	1:38	3:07	1:10	2:37	1:02

**Table 3:** Image quality in standard T2-weighted imaging (T2_S_) and deep learning reconstructed T2-weighted imaging (T2_DLR_).

Characteristics	Reader 1			Reader 2		
	T2_S_	T2_DLR_	*p*-Value	T2_S_	T2_DLR_	*p*-Value
Image noise axial	3 (3–3)	4 (4–4)	<0.001	3 (3–3)	4 (4–4)	<0.001
Image noise coronal	3 (3–3)	4 (4–4)	<0.001	3 (3–4)	4 (4–4)	<0.001
Image noise sagittal	3 (3–3)	4 (3–4)	<0.001	4 (3–4)	4 (3–4)	0.005
Artifacts axial	3 (3–4)	4 (4–4)	0.003	4 (3–4)	4 (4–4)	0.003
Artifacts coronal	3 (3–4)	4 (4–4)	0.002	3 (3–4)	4 (4–4)	0.014
Artifacts sagittal	3 (3–4)	4 (3–4)	0.002	3 (3–4)	4 (3–4)	0.011
Natural appearance axial	4 (4–4)	4 (4–4)	1	4 (4–4)	4 (4–4)	0.630
Natural appearance coronal	4 (4–4)	4 (4–4)	1	4 (4–4)	4 (4–4)	1
Natural appearance sagittal	4 (3–4)	4 (3–4)	1	4 (3–4)	4 (3–4)	1
Overall image quality axial	3 (3–4)	4 (4–4)	<0.001	3 (3–4)	4 (4–4)	<0.001
Overall image quality coronal	3 (3–4)	4 (4–4)	0.002	3 (3–4)	4 (4–4)	<0.001
Overall image quality sagittal	3 (3–4)	4 (3–4)	0.002	3 (3–4)	4 (3–4)	0.005
Diagnostic confidence	4 (3–4)	4 (4–4)	0.03	4 (3–4)	4 (4–4)	0.06

**Table 4:** T2 and PI-RADS scoring in standard T2-weighted imaging (T2_S_) and deep learning reconstructed T2-weighted imaging (T2_DLR_).

T2 and PI-RADS Scoring	Reader 1		Reader 2	
	T2_S_	T2_DLR_	T2_S_	T2_DLR_
T2 score				
1	0	0	0	0
2	21	21	21	21
3	14	13	13	14
4	15	16	16	15
5	10	10	10	10
PI-RADS score				
1	0	0	0	0
2	21	21	21	21
3	4	5	5	5
4	25	24	24	24
5	10	10	10	10

**Table 5:** Intra- and inter-reader agreement.

**Intra-Reader Agreement**	
Reader 1 T2 score	0.931
Reader 2 T2 score	0.905
Reader 1 PI-RADS score	0.960
Reader 2 PI-RADS score	0.946
**Inter-Reader Agreement**	
T2 score T2_S_	0.823
T2 score T2_DLR_	0.932
PI-RADS T2_S_	0.905
PI-RADS T2_DLR_	0.946

**Table 6:** Lesion size and conspicuity in standard T2-weighted imaging (T2_S_) and deep learning reconstructed T2-weighted imaging (T2_DLR_).

Characteristics	Reader 1			Reader 2		
	T2_S_	T2_DLR_	*p*-Value	T2_S_	T2_DLR_	*p*-Value
Lesion size (mm)	13 (10–16)	13 (11–16)	1	12 (10–16)	13 (10–17)	0.840
Lesion conspicuity	3 (3–4)	4 (4–4)	<0.001	3 (3–4)	4 (4–4)	0.001

## Data Availability

The datasets used and analyzed during the current study are available from the corresponding author on reasonable request.
